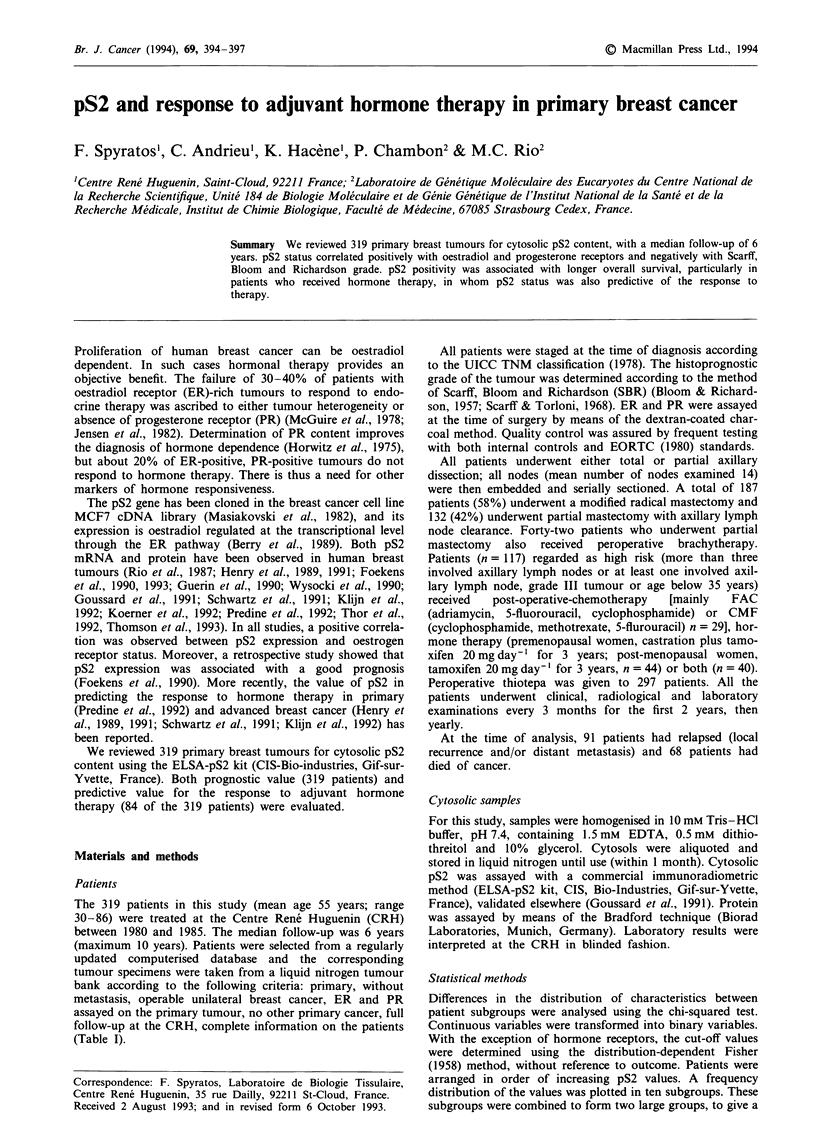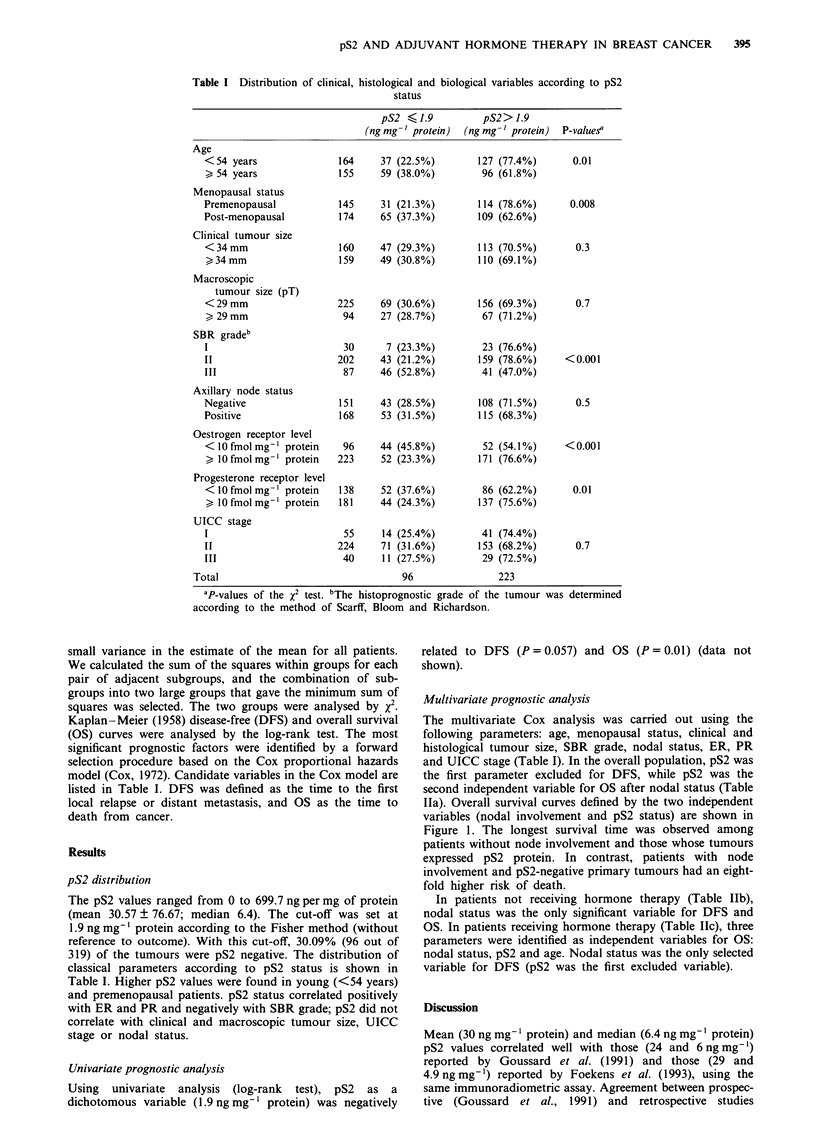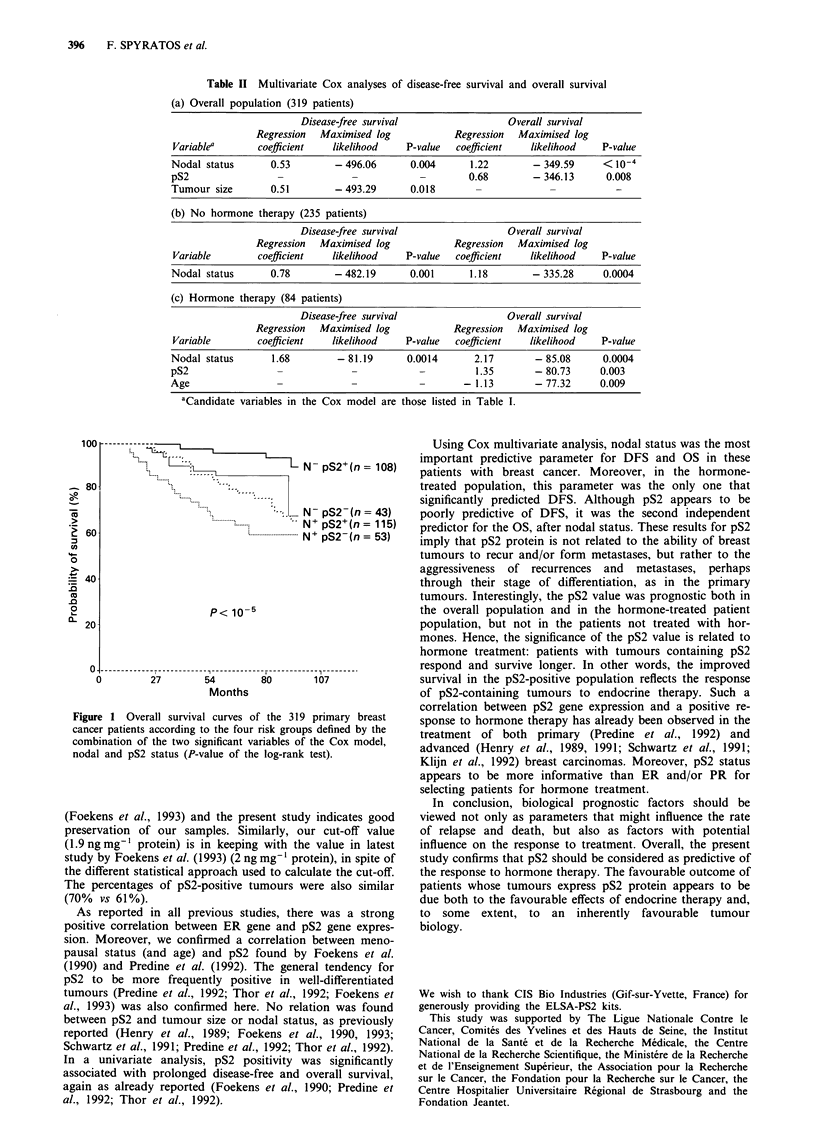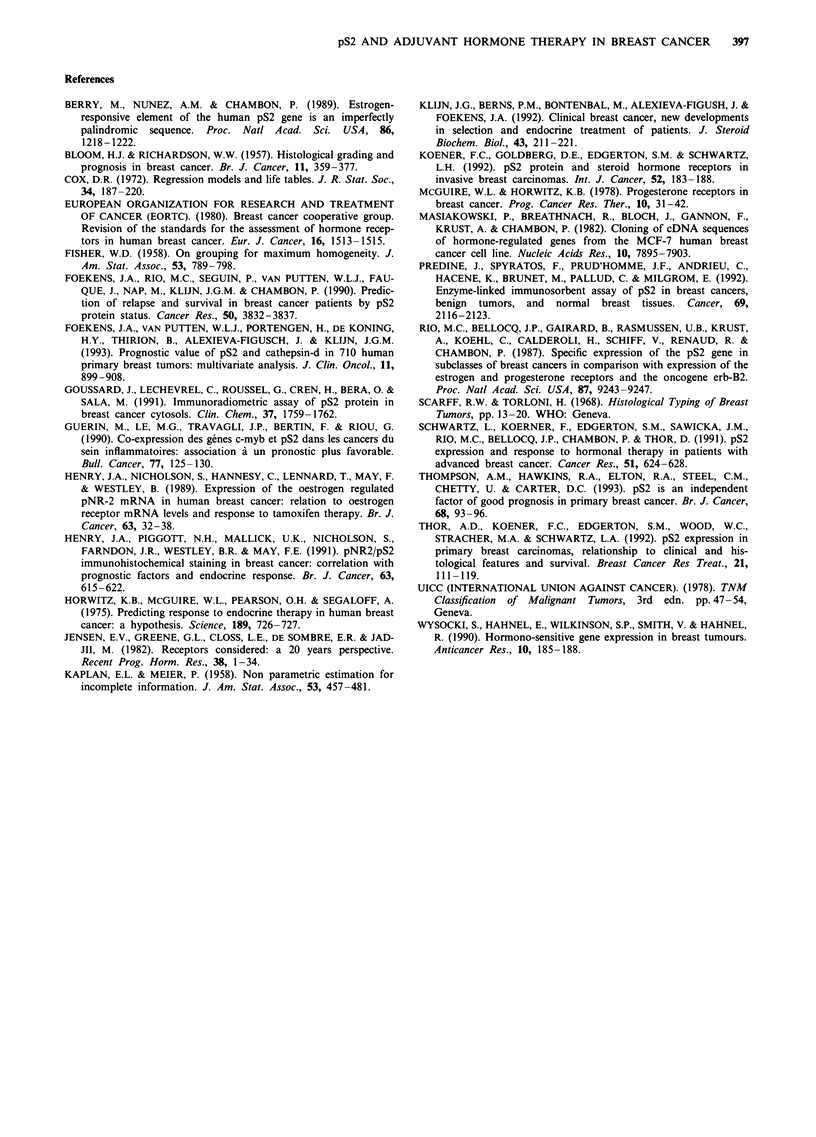# pS2 and response to adjuvant hormone therapy in primary breast cancer.

**DOI:** 10.1038/bjc.1994.73

**Published:** 1994-02

**Authors:** F. Spyratos, C. Andrieu, K. Hacène, P. Chambon, M. C. Rio

**Affiliations:** Centre René Huguenin, Saint-Cloud, France.

## Abstract

We reviewed 319 primary breast tumours for cytosolic pS2 content, with a median follow-up of 6 years. pS2 status correlated positively with oestradiol and progesterone receptors and negatively with Scarff, Bloom and Richardson grade. pS2 positivity was associated with longer overall survival, particularly in patients who received hormone therapy, in whom pS2 status was also predictive of the response to therapy.


Br. J. Cancer (1994), 69, 394-397 © Macmillan Press Ltd., 1994

### pS2 and response to adjuvant hormone therapy in primary breast cancer

F. Spyratos[1], C. Andrieu[1], K. Hacène[1], P. Chambon[2] & M.C. Rio[2]

[1]*Centre René Huguenin, Saint-Cloud, 92211 France;* [2]*Laboratoire de Génétique Moléculaire des Eucaryotes du Centre National de la Recherche Scientifique, Unité 184 de Biologie Moléculaire et de Génie Génétique de l'Institut National de la Santé et de la Recherche Médicale, Institut de Chimie Biologique, Faculté de Médecine, 67085 Strasbourg Cedex, France.*

**Summary** We reviewed 319 primary breast tumours for cytosolic pS2 content, with a median follow-up of 6 years. pS2 status correlated positively with oestradiol and progesterone receptors and negatively with Scarff, Bloom and Richardson grade. pS2 positivity was associated with longer overall survival, particularly in patients who received hormone therapy, in whom pS2 status was also predictive of the response to therapy.

Proliferation of human breast cancer can be oestradiol dependent. In such cases hormonal therapy provides an objective benefit. The failure of 30–40% of patients with oestradiol receptor (ER)-rich tumours to respond to endocrine therapy was ascribed to either tumour heterogeneity or absence of progesterone receptor (PR) (McGuire *et al.*, 1978; Jensen *et al.*, 1982). Determination of PR content improves the diagnosis of hormone dependence (Horwitz *et al.*, 1975), but about 20% of ER-positive, PR-positive tumours do not respond to hormone therapy. There is thus a need for other markers of hormone responsiveness.

The pS2 gene has been cloned in the breast cancer cell line MCF7 cDNA library (Masiakovski *et al.*, 1982), and its expression is oestradiol regulated at the transcriptional level through the ER pathway (Berry *et al.*, 1989). Both pS2 mRNA and protein have been observed in human breast tumours (Rio *et al.*, 1987; Henry *et al.*, 1989, 1991; Foekens *et al.*, 1990, 1993; Guerin *et al.*, 1990; Wysocki *et al.*, 1990; Goussard *et al.*, 1991; Schwartz *et al.*, 1991; Klijn *et al.*, 1992; Koerner *et al.*, 1992; Predine *et al.*, 1992; Thor *et al.*, 1992, Thomson *et al.*, 1993). In all studies, a positive correlation was observed between pS2 expression and oestrogen receptor status. Moreover, a retrospective study showed that pS2 expression was associated with a good prognosis (Foekens *et al.*, 1990). More recently, the value of pS2 in predicting the response to hormone therapy in primary (Predine *et al.*, 1992) and advanced breast cancer (Henry *et al.*, 1989, 1991; Schwartz *et al.*, 1991; Klijn *et al.*, 1992) has been reported.

We reviewed 319 primary breast tumours for cytosolic pS2 content using the ELSA-pS2 kit (CIS-Bio-industries, Gif-sur-Yvette, France). Both prognostic value (319 patients) and predictive value for the response to adjuvant hormone therapy (84 of the 319 patients) were evaluated.

### Materials and methods

### *Patients*

The 319 patients in this study (mean age 55 years; range 30–86) were treated at the Centre René Huguenin (CRH) between 1980 and 1985. The median follow-up was 6 years (maximum 10 years). Patients were selected from a regularly updated computerised database and the corresponding tumour specimens were taken from a liquid nitrogen tumour bank according to the following criteria: primary, without metastasis, operable unilateral breast cancer, ER and PR assayed on the primary tumour, no other primary cancer, full follow-up at the CRH, complete information on the patients (Table I).

Correspondence: F. Spyratos, Laboratoire de Biologie Tissulaire, Centre René Huguenin, 35 rue Dailly, 92211 St-Cloud, France.
Received 2 August 1993; and in revised form 6 October 1993.

All patients were staged at the time of diagnosis according to the UICC TNM classification (1978). The histoprognostic grade of the tumour was determined according to the method of Scarff, Bloom and Richardson (SBR) (Bloom & Richardson, 1957; Scarff & Torloni, 1968). ER and PR were assayed at the time of surgery by means of the dextran-coated charcoal method. Quality control was assured by frequent testing with both internal controls and EORTC (1980) standards.

All patients underwent either total or partial axillary dissection; all nodes (mean number of nodes examined 14) were then embedded and serially sectioned. A total of 187 patients (58%) underwent a modified radical mastectomy and 132 (42%) underwent partial mastectomy with axillary lymph node clearance. Forty-two patients who underwent partial mastectomy also received peroperative brachytherapy. Patients ($n = 117$) regarded as high risk (more than three involved axillary lymph nodes or at least one involved axillary lymph node, grade III tumour or age below 35 years) received post-operative-chemotherapy [mainly FAC (adriamycin, 5-fluorouracil, cyclophosphamide) or CMF (cyclophosphamide, methotrexate, 5-flurouracil) $n = 29$], hormone therapy (premenopausal women, castration plus tamoxifen 20 mg day$^{-1}$ for 3 years; post-menopausal women, tamoxifen 20 mg day$^{-1}$ for 3 years, $n = 44$) or both ($n = 40$). Peroperative thiotepa was given to 297 patients. All the patients underwent clinical, radiological and laboratory examinations every 3 months for the first 2 years, then yearly.

At the time of analysis, 91 patients had relapsed (local recurrence and/or distant metastasis) and 68 patients had died of cancer.

### *Cytosolic samples*

For this study, samples were homogenised in 10 mM Tris–HCl buffer, pH 7.4, containing 1.5 mM EDTA, 0.5 mM dithiothreitol and 10% glycerol. Cytosols were aliquoted and stored in liquid nitrogen until use (within 1 month). Cytosolic pS2 was assayed with a commercial immunoradiometric method (ELSA-pS2 kit, CIS, Bio-Industries, Gif-sur-Yvette, France), validated elsewhere (Goussard *et al.*, 1991). Protein was assayed by means of the Bradford technique (Biorad Laboratories, Munich, Germany). Laboratory results were interpreted at the CRH in blinded fashion.

### *Statistical methods*

Differences in the distribution of characteristics between patient subgroups were analysed using the chi-squared test. Continuous variables were transformed into binary variables. With the exception of hormone receptors, the cut-off values were determined using the distribution-dependent Fisher (1958) method, without reference to outcome. Patients were arranged in order of increasing pS2 values. A frequency distribution of the values was plotted in ten subgroups. These subgroups were combined to form two large groups, to give a

**Table I** Distribution of clinical, histological and biological variables according to pS2 status

| | | *pS2 ⩽1.9 (ng mg$^{-1}$ protein)* | *pS2>1.9 (ng mg$^{-1}$ protein)* | P-*values*[a] |
|---|---|---|---|---|
| Age | | | | |
| <54 years | 164 | 37 (22.5%) | 127 (77.4%) | 0.01 |
| ⩾54 years | 155 | 59 (38.0%) | 96 (61.8%) | |
| Menopausal status | | | | |
| Premenopausal | 145 | 31 (21.3%) | 114 (78.6%) | 0.008 |
| Post-menopausal | 174 | 65 (37.3%) | 109 (62.6%) | |
| Clinical tumour size | | | | |
| <34 mm | 160 | 47 (29.3%) | 113 (70.5%) | 0.3 |
| ⩾34 mm | 159 | 49 (30.8%) | 110 (69.1%) | |
| Macroscopic tumour size (pT) | | | | |
| <29 mm | 225 | 69 (30.6%) | 156 (69.3%) | 0.7 |
| ⩾29 mm | 94 | 27 (28.7%) | 67 (71.2%) | |
| SBR grade[b] | | | | |
| I | 30 | 7 (23.3%) | 23 (76.6%) | |
| II | 202 | 43 (21.2%) | 159 (78.6%) | <0.001 |
| III | 87 | 46 (52.8%) | 41 (47.0%) | |
| Axillary node status | | | | |
| Negative | 151 | 43 (28.5%) | 108 (71.5%) | 0.5 |
| Positive | 168 | 53 (31.5%) | 115 (68.3%) | |
| Oestrogen receptor level | | | | |
| <10 fmol mg$^{-1}$ protein | 96 | 44 (45.8%) | 52 (54.1%) | <0.001 |
| ⩾10 fmol mg$^{-1}$ protein | 223 | 52 (23.3%) | 171 (76.6%) | |
| Progesterone receptor level | | | | |
| <10 fmol mg$^{-1}$ protein | 138 | 52 (37.6%) | 86 (62.2%) | 0.01 |
| ⩾10 fmol mg$^{-1}$ protein | 181 | 44 (24.3%) | 137 (75.6%) | |
| UICC stage | | | | |
| I | 55 | 14 (25.4%) | 41 (74.4%) | |
| II | 224 | 71 (31.6%) | 153 (68.2%) | 0.7 |
| III | 40 | 11 (27.5%) | 29 (72.5%) | |
| Total | | 96 | 223 | |

[a]*P*-values of the $\chi^2$ test. [b]The histoprognostic grade of the tumour was determined according to the method of Scarff, Bloom and Richardson.

small variance in the estimate of the mean for all patients. We calculated the sum of the squares within groups for each pair of adjacent subgroups, and the combination of subgroups into two large groups that gave the minimum sum of squares was selected. The two groups were analysed by $\chi^2$. Kaplan–Meier (1958) disease-free (DFS) and overall survival (OS) curves were analysed by the log-rank test. The most significant prognostic factors were identified by a forward selection procedure based on the Cox proportional hazards model (Cox, 1972). Candidate variables in the Cox model are listed in Table I. DFS was defined as the time to the first local relapse or distant metastasis, and OS as the time to death from cancer.

### Results

### *pS2 distribution*

The pS2 values ranged from 0 to 699.7 ng per mg of protein (mean 30.57 ± 76.67; median 6.4). The cut-off was set at 1.9 ng mg$^{-1}$ protein according to the Fisher method (without reference to outcome). With this cut-off, 30.09% (96 out of 319) of the tumours were pS2 negative. The distribution of classical parameters according to pS2 status is shown in Table I. Higher pS2 values were found in young (<54 years) and premenopausal patients. pS2 status correlated positively with ER and PR and negatively with SBR grade; pS2 did not correlate with clinical and macroscopic tumour size, UICC stage or nodal status.

### *Univariate prognostic analysis*

Using univariate analysis (log-rank test), pS2 as a dichotomous variable (1.9 ng mg$^{-1}$ protein) was negatively related to DFS ($P = 0.057$) and OS ($P = 0.01$) (data not shown).

### *Multivariate prognostic analysis*

The multivariate Cox analysis was carried out using the following parameters: age, menopausal status, clinical and histological tumour size, SBR grade, nodal status, ER, PR and UICC stage (Table I). In the overall population, pS2 was the first parameter excluded for DFS, while pS2 was the second independent variable for OS after nodal status (Table IIa). Overall survival curves defined by the two independent variables (nodal involvement and pS2 status) are shown in Figure 1. The longest survival time was observed among patients without node involvement and those whose tumours expressed pS2 protein. In contrast, patients with node involvement and pS2-negative primary tumours had an eight-fold higher risk of death.

In patients not receiving hormone therapy (Table IIb), nodal status was the only significant variable for DFS and OS. In patients receiving hormone therapy (Table IIc), three parameters were identified as independent variables for OS: nodal status, pS2 and age. Nodal status was the only selected variable for DFS (pS2 was the first excluded variable).

### Discussion

Mean (30 ng mg$^{-1}$ protein) and median (6.4 ng mg$^{-1}$ protein) pS2 values correlated well with those (24 and 6 ng mg$^{-1}$) reported by Goussard *et al.* (1991) and those (29 and 4.9 ng mg$^{-1}$) reported by Foekens *et al.* (1993), using the same immunoradiometric assay. Agreement between prospective (Goussard *et al.*, 1991) and retrospective studies

**Table II** Multivariate Cox analyses of disease-free survival and overall survival

(a) Overall population (319 patients)

| | Disease-free survival | | | Overall survival | | |
|---|---|---|---|---|---|---|
| *Variable*[a] | *Regression coefficient* | *Maximised log likelihood* | P-*value* | *Regression coefficient* | *Maximised log likelihood* | P-*value* |
| Nodal status | 0.53 | − 496.06 | 0.004 | 1.22 | − 349.59 | $<10^{-4}$ |
| pS2 | – | – | – | 0.68 | − 346.13 | 0.008 |
| Tumour size | 0.51 | − 493.29 | 0.018 | – | – | – |

(b) No hormone therapy (235 patients)

| | Disease-free survival | | | Overall survival | | |
|---|---|---|---|---|---|---|
| *Variable* | *Regression coefficient* | *Maximised log likelihood* | P-*value* | *Regression coefficient* | *Maximised log likelihood* | P-*value* |
| Nodal status | 0.78 | − 482.19 | 0.001 | 1.18 | − 335.28 | 0.0004 |

(c) Hormone therapy (84 patients)

| | Disease-free survival | | | Overall survival | | |
|---|---|---|---|---|---|---|
| *Variable* | *Regression coefficient* | *Maximised log likelihood* | P-*value* | *Regression coefficient* | *Maximised log likelihood* | P-*value* |
| Nodal status | 1.68 | − 81.19 | 0.0014 | 2.17 | − 85.08 | 0.0004 |
| pS2 | – | – | – | 1.35 | − 80.73 | 0.003 |
| Age | – | – | – | − 1.13 | − 77.32 | 0.009 |

[a]Candidate variables in the Cox model are those listed in Table I.

**Figure 1** Overall survival curves of the 319 primary breast cancer patients according to the four risk groups defined by the combination of the two significant variables of the Cox model, nodal and pS2 status (*P*-value of the log-rank test).

(Foekens *et al.*, 1993) and the present study indicates good preservation of our samples. Similarly, our cut-off value (1.9 ng mg$^{-1}$ protein) is in keeping with the value in latest study by Foekens *et al.* (1993) (2 ng mg$^{-1}$ protein), in spite of the different statistical approach used to calculate the cut-off. The percentages of pS2-positive tumours were also similar (70% *vs* 61%).

As reported in all previous studies, there was a strong positive correlation between ER gene and pS2 gene expression. Moreover, we confirmed a correlation between menopausal status (and age) and pS2 found by Foekens *et al.* (1990) and Predine *et al.* (1992). The general tendency for pS2 to be more frequently positive in well-differentiated tumours (Predine *et al.*, 1992; Thor *et al.*, 1992; Foekens *et al.*, 1993) was also confirmed here. No relation was found between pS2 and tumour size or nodal status, as previously reported (Henry *et al.*, 1989; Foekens *et al.*, 1990, 1993; Schwartz *et al.*, 1991; Predine *et al.*, 1992; Thor *et al.*, 1992). In a univariate analysis, pS2 positivity was significantly associated with prolonged disease-free and overall survival, again as already reported (Foekens *et al.*, 1990; Predine *et al.*, 1992; Thor *et al.*, 1992).

Using Cox multivariate analysis, nodal status was the most important predictive parameter for DFS and OS in these patients with breast cancer. Moreover, in the hormone-treated population, this parameter was the only one that significantly predicted DFS. Although pS2 appears to be poorly predictive of DFS, it was the second independent predictor for the OS, after nodal status. These results for pS2 imply that pS2 protein is not related to the ability of breast tumours to recur and/or form metastases, but rather to the aggressiveness of recurrences and metastases, perhaps through their stage of differentiation, as in the primary tumours. Interestingly, the pS2 value was prognostic both in the overall population and in the hormone-treated patient population, but not in the patients not treated with hormones. Hence, the significance of the pS2 value is related to hormone treatment: patients with tumours containing pS2 respond and survive longer. In other words, the improved survival in the pS2-positive population reflects the response of pS2-containing tumours to endocrine therapy. Such a correlation between pS2 gene expression and a positive response to hormone therapy has already been observed in the treatment of both primary (Predine *et al.*, 1992) and advanced (Henry *et al.*, 1989, 1991; Schwartz *et al.*, 1991; Klijn *et al.*, 1992) breast carcinomas. Moreover, pS2 status appears to be more informative than ER and/or PR for selecting patients for hormone treatment.

In conclusion, biological prognostic factors should be viewed not only as parameters that might influence the rate of relapse and death, but also as factors with potential influence on the response to treatment. Overall, the present study confirms that pS2 should be considered as predictive of the response to hormone therapy. The favourable outcome of patients whose tumours express pS2 protein appears to be due both to the favourable effects of endocrine therapy and, to some extent, to an inherently favourable tumour biology.

We wish to thank CIS Bio Industries (Gif-sur-Yvette, France) for generously providing the ELSA-PS2 kits.

This study was supported by The Ligue Nationale Contre le Cancer, Comités des Yvelines et des Hauts de Seine, the Institut National de la Santé et de la Recherche Médicale, the Centre National de la Recherche Scientifique, the Ministère de la Recherche et de l'Enseignement Supérieur, the Association pour la Recherche sur le Cancer, the Fondation pour la Recherche sur le Cancer, the Centre Hospitalier Universitaire Régional de Strasbourg and the Fondation Jeantet.

### References

BERRY, M., NUNEZ, A.M. & CHAMBON, P. (1989). Estrogen-responsive element of the human pS2 gene is an imperfectly palindromic sequence. *Proc. Natl Acad. Sci. USA*, **86**, 1218–1222.

BLOOM, H.J. & RICHARDSON, W.W. (1957). Histological grading and prognosis in breast cancer. *Br. J. Cancer*, **11**, 359–377.

COX, D.R. (1972). Regression models and life tables. *J. R. Stat. Soc.*, **34**, 187–220.

EUROPEAN ORGANIZATION FOR RESEARCH AND TREATMENT OF CANCER (EORTC). (1980). Breast cancer cooperative group. Revision of the standards for the assessment of hormone receptors in human breast cancer. *Eur. J. Cancer*, **16**, 1513–1515.

FISHER, W.D. (1958). On grouping for maximum homogeneity. *J. Am. Stat. Assoc.*, **53**, 789–798.

FOEKENS, J.A., RIO, M.C., SEGUIN, P., VAN PUTTEN, W.L.J., FAUQUE, J., NAP, M., KLIJN, J.G.M. & CHAMBON, P. (1990). Prediction of relapse and survival in breast cancer patients by pS2 protein status. *Cancer Res.*, **50**, 3832–3837.

FOEKENS, J.A., VAN PUTTEN, W.L.J., PORTENGEN, H., DE KONING, H.Y., THIRION, B., ALEXIEVA-FIGUSCH, J. & KLIJN, J.G.M. (1993). Prognostic value of pS2 and cathepsin-d in 710 human primary breast tumors: multivariate analysis. *J. Clin. Oncol.*, **11**, 899–908.

GOUSSARD, J., LECHEVREL, C., ROUSSEL, G., CREN, H., BERA, O. & SALA, M. (1991). Immunoradiometric assay of pS2 protein in breast cancer cytosols. *Clin. Chem.*, **37**, 1759–1762.

GUERIN, M., LE, M.G., TRAVAGLI, J.P., BERTIN, F. & RIOU, G. (1990). Co-expression des génes c-myb et pS2 dans les cancers du sein inflammatoires: association à un pronostic plus favorable. *Bull. Cancer*, **77**, 125–130.

HENRY, J.A., NICHOLSON, S., HANNESY, C., LENNARD, T., MAY, F. & WESTLEY, B. (1989). Expression of the oestrogen regulated pNR-2 mRNA in human breast cancer: relation to oestrogen receptor mRNA levels and response to tamoxifen therapy. *Br. J. Cancer*, **63**, 32–38.

HENRY, J.A., PIGGOTT, N.H., MALLICK, U.K., NICHOLSON, S., FARNDON, J.R., WESTLEY, B.R. & MAY, F.E. (1991). pNR2/pS2 immunohistochemical staining in breast cancer: correlation with prognostic factors and endocrine response. *Br. J. Cancer*, **63**, 615–622.

HORWITZ, K.B., MCGUIRE, W.L., PEARSON, O.H. & SEGALOFF, A. (1975). Predicting response to endocrine therapy in human breast cancer: a hypothesis. *Science*, **189**, 726–727.

JENSEN, E.V., GREENE, G.L., CLOSS, L.E., DE SOMBRE, E.R. & JADJII, M. (1982). Receptors considered: a 20 years perspective. *Recent Prog. Horm. Res.*, **38**, 1–34.

KAPLAN, E.L. & MEIER, P. (1958). Non parametric estimation for incomplete information. *J. Am. Stat. Assoc.*, **53**, 457–481.

KLIJN, J.G., BERNS, P.M., BONTENBAL, M., ALEXIEVA-FIGUSH, J. & FOEKENS, J.A. (1992). Clinical breast cancer, new developments in selection and endocrine treatment of patients. *J. Steroid Biochem. Biol.*, **43**, 211–221.

KOENER, F.C., GOLDBERG, D.E., EDGERTON, S.M. & SCHWARTZ, L.H. (1992). pS2 protein and steroid hormone receptors in invasive breast carcinomas. *Int. J. Cancer*, **52**, 183–188.

MCGUIRE, W.L. & HORWITZ, K.B. (1978). Progesterone receptors in breast cancer. *Prog. Cancer Res. Ther.*, **10**, 31–42.

MASIAKOWSKI, P., BREATHNACH, R., BLOCH, J., GANNON, F., KRUST, A. & CHAMBON, P. (1982). Cloning of cDNA sequences of hormone-regulated genes from the MCF-7 human breast cancer cell line. *Nucleic Acids Res.*, **10**, 7895–7903.

PREDINE, J., SPYRATOS, F., PRUD'HOMME, J.F., ANDRIEU, C., HACENE, K., BRUNET, M., PALLUD, C. & MILGROM, E. (1992). Enzyme-linked immunosorbent assay of pS2 in breast cancers, benign tumors, and normal breast tissues. *Cancer*, **69**, 2116–2123.

RIO, M.C., BELLOCQ, J.P., GAIRARD, B., RASMUSSEN, U.B., KRUST, A., KOEHL, C., CALDEROLI, H., SCHIFF, V., RENAUD, R. & CHAMBON, P. (1987). Specific expression of the pS2 gene in subclasses of breast cancers in comparison with expression of the estrogen and progesterone receptors and the oncogene erb-B2. *Proc. Natl Acad. Sci. USA*, **87**, 9243–9247.

SCARFF, R.W. & TORLONI, H. (1968). *Histological Typing of Breast Tumors*, pp. 13–20. WHO: Geneva.

SCHWARTZ, L., KOERNER, F., EDGERTON, S.M., SAWICKA, J.M., RIO, M.C., BELLOCQ, J.P., CHAMBON, P. & THOR, D. (1991). pS2 expression and response to hormonal therapy in patients with advanced breast cancer. *Cancer Res.*, **51**, 624–628.

THOMPSON, A.M., HAWKINS, R.A., ELTON, R.A., STEEL, C.M., CHETTY, U. & CARTER, D.C. (1993). pS2 is an independent factor of good prognosis in primary breast cancer. *Br. J. Cancer*, **68**, 93–96.

THOR, A.D., KOENER, F.C., EDGERTON, S.M., WOOD, W.C., STRACHER, M.A. & SCHWARTZ, L.A. (1992). pS2 expression in primary breast carcinomas, relationship to clinical and histological features and survival. *Breast Cancer Res Treat.*, **21**, 111–119.

UICC (INTERNATIONAL UNION AGAINST CANCER). (1978). *TNM Classification of Malignant Tumors*, 3rd edn. pp. 47–54, Geneva.

WYSOCKI, S., HAHNEL, E., WILKINSON, S.P., SMITH, V. & HAHNEL, R. (1990). Hormono-sensitive gene expression in breast tumours. *Anticancer Res.*, **10**, 185–188.